# Skeletal Muscle and COVID-19: The Potential Involvement of Bioactive Sphingolipids

**DOI:** 10.3390/biomedicines10051068

**Published:** 2022-05-04

**Authors:** Elisabetta Meacci, Federica Pierucci, Mercedes Garcia-Gil

**Affiliations:** 1 Unit of Biochemical Sciences and Molecular Biology, Department of Experimental and Clinical Biomedical Sciences “Mario Serio”, University of Florence, Viale GB Morgagni 50, 50121 Florence, Italy; federica.pierucci@unifi.it; 2 Interuniversity Institute of Myology, University of Florence, 50121 Florence, Italy; 3 Unit of Physiology, Department of Biology, University of Pisa, Via S. Zeno 31, 56127 Pisa, Italy; mercedes.garcia@unipi.it; 4 Interdepartmental Research Center “Nutraceuticals and Food for Health”, University of Pisa, 56127 Pisa, Italy

**Keywords:** COVID-19, SARS-CoV-2, sphingolipids, sphingosine 1-phosphate, ceramide, sphingosine 1-phosphate receptors, skeletal muscle, myasthenia gravis, multiple sclerosis, amyotrophic lateral sclerosis

## Abstract

SARS-CoV-2 virus infection is the cause of the coronavirus disease 2019 (COVID-19), which is still spreading over the world. The manifestation of this disease can range from mild to severe and can be limited in time (weeks) or persist for months in about 30–50% of patients. COVID-19 is considered a multiple organ dysfunction syndrome and the musculoskeletal system manifestations are beginning to be considered of absolute importance in both COVID-19 patients and in patients recovering from the SARS-CoV-2 infection. Musculoskeletal manifestations of COVID-19 and other coronavirus infections include loss of muscle mass, muscle weakness, fatigue or myalgia, and muscle injury. The molecular mechanisms by which SARS-CoV-2 can cause damage to skeletal muscle (SkM) cells are not yet well understood. Sphingolipids (SLs) represent an important class of eukaryotic lipids with structural functions as well as bioactive molecules able to modulate crucial processes, including inflammation and viral infection. In the last two decades, several reports have highlighted the role of SLs in modulating SkM cell differentiation, regeneration, aging, response to insulin, and contraction. This review summarizes the consequences of SARS-CoV-2 infection on SkM and the potential involvement of SLs in the tissue responses to virus infection. In particular, we highlight the role of sphingosine 1-phosphate signaling in order to aid the prediction of novel targets for preventing and/or treating acute and long-term musculoskeletal manifestations of virus infection in COVID-19.

## 1. Introduction

Coronavirus disease 2019 (COVID-19) is a worldwide disease due to severe acute respiratory syndrome coronavirus 2 (SARS-CoV-2) infection. The manifestation of COVID-19 can range from mild to severe [1,2,3,4]: the majority of cases show mild symptomatic COVID-19 characterized by fever, shortness of breath, gastrointestinal dysfunctions, headaches, and a loss of taste and smell and, sometimes, mild pneumonia. Other COVID-19 patients manifest more severe symptoms mainly respiratory failure that requires mechanical ventilation support. Although these symptoms are common in the different variants caused by mutations in SARS-CoV-2 [4,5], the prevalence of symptoms caused by the variants might differ. COVID-19 is now considered a multiple organ dysfunction syndrome since SARS-CoV-2 is able to invade multiple organs and promote systemic inflammation [6,7,8]. Moreover, the comorbidities such as diabetes mellitus, hypertension, cardiovascular disease, or chronic obstructive pulmonary disease are also risk factors for severity in patients with COVID-19 [9,10]. Notably, many patients infected with SARS-CoV-2 (approximately 30–50% of patients) develop a post-acute syndrome, which persist months after the initial infection defined “long COVID-19” [11,12,13]. The musculoskeletal system manifestations are beginning to be considered of absolute importance, not only in COVID-19 patients, but also in patients recovering from the SARS-CoV-2 infection and in patients who had severe acute respiratory syndrome (SARS) [14,15,16,17]. Musculoskeletal manifestations of COVID-19 and other coronavirus infections include loss of muscle mass, muscle weakness, fatigue or myalgia, and muscle injury [18,19,20]. However, the mechanisms by which SARS-CoV-2 can cause damage to SkM cells are not yet understood.

The coronaviruses (CoVs) belonging to the Orthocoronaviridae subfamily and to Coronaviridae family consist of four genera, including α/β/γ/δ-CoV and only α and β infect mammals. Other strains, capable of infecting humans, are part of the same family of SARS-CoV-2, which includes the more pathogenic viruses SARS-CoV-1, responsible for SARS reviewed in [21]. SARS-CoV-2 are single and positive-stranded RNA viruses [21], enveloped with a lipid membrane enriched with structural proteins, including the spike protein, which is responsible for the typical appearance of a solar corona. Spike protein protrudes from the virus membrane and it is essential for host receptor binding and cell tropism and, therefore, for virus infection. The mechanism of infection of SARS-CoV-1 and SARS-CoV-2 are similar. They enter cells via the angiotensin-converting enzyme 2 (ACE2) receptor highly expressed in humans mainly in the respiratory and intestinal tract, and less in other tissues, such as SkM [22,23]. Following receptor binding, the viral Spike protein is proteolytically cleaved by the transmembrane serine protease 2 (TMPRSS2) [23], which is indispensable for viral spread and pathogenesis permitting mixing of viral and human membranes and release of viral RNA into the cytoplasm [24]. In this cellular compartment, the viral RNA replication begins with translation of the replicase-polymerase gene and assembly, which transcribes the genomic regions to structural proteins and ultimately leads to the assembly of virions, then released from infected cells by exocytosis.

**Figure 1:**
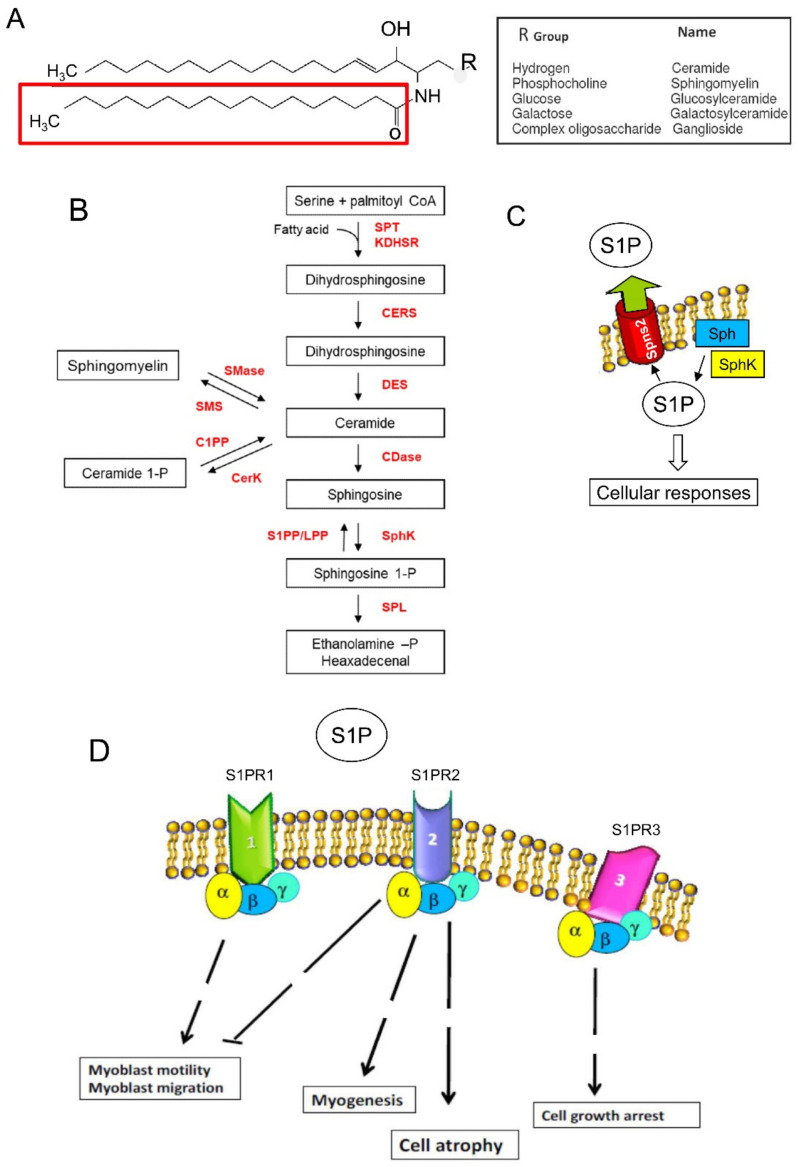
Sphingolipid metabolism and sphingosine-1-phosphate (Sphingosine 1-P) signaling. (**A**) General sphingolipid structure. Sphingolipids are composed of a sphingosine backbone linked to a fatty acid. (**B**) Sphingomyelin cycle or de novo sphingolipid synthesis leading to ceramide involving serine palmitoyl transferase (SPT), 3-keto dihydrosphingosine reductase (KDHSR), ceramide synthase (CERS), and desaturase (DES). Ceramide is converted reversibly to sphingosine by ceramidase (CDase) or phosphorylated to ceramide-1-phosphate (Ceramide 1-P) by ceramide kinase (CerK) activity and dephosphorylated by ceramide-1P phosphatase (C1PP). S1P is synthesized from sphingosine by the sphingosine kinases (SphK) and irreversibly cleaved by S1P lyase (SPL), which generates hexadecenal and phosphoethanolamine (ethanolamine -P). S1P is also a substrate of specific S1P phosphatases (S1PP) or lipid phosphate phosphohydrolase (LPP). Sphingomyelin synthases (SMS) transfer a phosphorylcholine group from phosphatidylcholine to ceramide, generating diacylglycerol and sphingomyelin. Sphingomyelinases (SMase) catalyze the hydrolysis of sphingomyelin, leading to the generation of ceramide and phosphorylcholine. (**C**) S1P produced inside the cell can be transported in the intercellular space by an ATP-binding cassette transporter named spinster homolog 2 (Spns2). (**D**) As a ligand, S1P acts as autocrine and paracrine factors triggering specific signaling pathways by interacting with S1P specific heterotrimeric GTP binding protein-coupled receptors, named S1PR. Three among five subtypes of S1PRs, S1PR-1, -2, and -3, are expressed in skeletal muscle cells and regulate through different steps (broken lines) specific biological functions. The scheme exemplifies the main roles played by S1PR activation in skeletal muscle cells.

The Coronavirus infection can lead to an acute immune response. Infected pneumocytes, through the release of pathogen- and damage-associated molecular proteins activate inflammatory alveolar- and monocyte-derived macrophages, which initiate the acute inflammatory cascade release of several pro-inflammatory mediators [25]. Most COVID-19 patients suffer a so-called “cytokine storm syndrome,” characterized by the release from cells of cytokines and signaling molecules such as the C-X-C motif chemokine 10 (CXCL10), interferon gamma (IFN-γ), interleukin 1 beta (IL-1β), IL-6, IL-8, IL-17, and tumor necrosis factor alpha (TNF-α) [26,27]. Sphingolipids (SLs), one of the major classes of eukaryotic lipids, are composed of a sphingoid backbone, which can be modified by phosphorylation, glycosylation, and acylation (Figure 1). They have an essential structural role, and are also able to modulate crucial processes, such as inflammation and viral infection, including those related to SARS-CoV-2 [28,29,30]. This is not surprising since sphingolipids regulate cell fate (cell proliferation, differentiation, survival, senescence, autophagy, and apoptosis [27,31,32,33]. In particular, a rheostat system has been described underlining the importance of the ratio between the unphosphorylated and phosphorylated forms of the bioactive sphingoids [34,35]. In fact, ceramide and sphingosine-1-phosphate (S1P) act in opposite manners: ceramide mainly controls cell growth arrest, senescence, and cell death, whereas S1P regulates cell proliferation, migration, and survival. Several subclasses of ceramide are increased in plasma of SARS-CoV-2 patients and this increase is higher in those with respiratory distress [36]. SLs have an integral role in inflammation. For example, the bioactive SLs produce pro-inflammatory prostaglandins due to the activation of pro-inflammatory transcription factors in different cell types and induction of cyclooxygenase-2 [27]. The S1P-S1P receptor signaling system modulates lymphocyte trafficking and maintenance of vascular integrity, thereby contributing to the regulation of inflammation [37]. Moreover, S1P also acts as an intracellular second messenger by direct stimulation of intracellular signaling proteins involved in both inflammation and survival [38]. In the last two decades, several reports have highlighted the role of SLs in modulating SkM cell differentiation, regeneration, aging, response to insulin, and contraction [31,39]. In vivo and in vitro studies have demonstrated that S1P, acting as ligand of specific S1P receptors (S1PRs), plays a major role in the activation of satellite cells, myogenesis, and cell regeneration [31,40,41,42,43,44,45,46,47] (Figure 1C,D).

Interestingly, the pattern expression of S1PR changes during myoblast differentiation and in Dexamethasone-induced cell atrophy and in SkM of C26-cachectic mice [41,43,46]. S1P could also protect skeletal muscle tissue against eccentric contraction-induced damage [48] and denervation [49]. SL metabolism impairment controls SkM fatigue [50,51]. Contrarily, ceramide appears to negatively control myogenesis [52,53] and myocyte size in a mouse model of cancer-induced cachexia [54,55]. Notably, all these considerations reinforce the notion that the ceramide/S1P rheostat play a role also in SkM differentiation and muscle mass and fatigue regulation. Interestingly, Teo et al., (2018) [56] reported that the treatment with Fingolimod, a modulator of S1PR (see 4.1), abrogates Chikungunya-virusinduced arthralgia and pain. The potential efficacy of Fingolimod in SARS-CoV-2 infection has also been suggested [57]. Therefore, the purpose of this article was to summarize our knowledge on the consequences of SARS-CoV-2 infection on SkM and the potential involvement of SLs on SkM fate. In particular, we highlight the role of S1P signaling in order to aid the prediction of novel targets for preventing and/or treating acute and long-term musculoskeletal consequences of COVID-19 infection.

## 2. Skeletal Muscle Manifestations in COVID-19 Patients

COVID-19 is associated with SkM complications, which comprise myalgia, weakness, mass loss, myositis, and rhabdomyolysis [58,59,60,61,62]. SkM damage has been also reported following infection of several viruses. Leung et al., reported in post-mortem SkM tissue of patients affected by SARS, a widespread muscle fiber atrophy, fiber necrosis, myofibril disarray, and loss of Z-discs [16]. A reduced hand grip strength (32%) was also reported in SARS patients [63]. Similarly, SkM manifestations, such as inclusion-body myositis, chronic fatigue, and opportunistic infections are one of the first symptoms associated with human immunodeficiency virus (HIV) infection [64]. Numerous cases of acute myopathy and/or rhabdomyolysis have been also reported, following the outbreak of pandemic influenza A (H1N1) in 2009 [65]. In the next sections, we briefly revise the acute and long-term manifestations of SkM in COVID-19 patients.

### 2.1. Acute Manifestations

The effects of SARS-CoV-2 infection on SkM cells, at cellular level, are not well clarified. One of the first studies conducted on 214 COVID-19 patients hospitalized in Wuhan, indicated that 8.9% of patients presented peripheral nerve disease, and 7% had muscular injuries. Moreover, among patients with severe COVID-19, 19.3% had evidence of muscle injury [58]. However, up to 19.4% of patients present with myalgia and elevated levels of creatine kinase (CK) (>200 U/L), suggesting SkM injury. Similar results were found during autopsies [20,66].

In vitro studies have shown that SARS-CoV-2 can cause myofibrillar fragmentation into individual sarcomeres of human induced pluripotent stem cell (iPSC)-derived heart cells [67]. As noted, electron micrographs of SkM tissue from patients with COVID-19 revealed myofibril disarray and Z disc streaming, accompanied with disruption of force transmission [68]. In post-mortem COVID-19 specimens, muscle bundles contained few fibers with marked atrophy, degenerated fibers showed cytoplasm devoid of striations, and necrobiotic fibers were observed with few lymphocytes rimming or invading them [69]. Interestingly, in a clinical case, substantial reductions in vascular, muscular, and mitochondrial functions were observed along with an elevation in IL-10 before symptom occurrence [70]. Moreover, Hooper et al., (2021) [71] have detected in SkM fibrin microthrombi, perimysial microhemorrhages, and adjacent muscle fiber vacuolar degeneration and necrosis in an autoptic specimen. More inflammatory features are shown in SkM than cardiac muscles, especially in patients with chronic COVID-19 disease [66,72]. In fact, Aschman et al., (2021) [66] reported that 26 out of 43 individuals with severe COVID-19 disease showed signs of myositis from mild to severe. Inflammation of SkM was associated with the duration of illness. Similarly, SkM dysfunctions, structural alterations, and myalgia were also described in patients with SARS [16,63,73]. Biomarkers of inflammation and SkM injury were significantly elevated in patients with both less severe and fatal COVID-19. In particular, SkM damage markers, such as CK, and other factors, such as C reactive protein, lactate dehydrogenase, and ferritin, have been observed at increased levels in patients with COVID-19-related myositis and rhabdomyolysis [66,74]. Furthermore, characteristic protein and metabolite changes in the sera of severe COVID-19 patients within a few days of hospital admission have been reported. This first study [75] and others (see below) performed later, will be useful in the selection of potential blood biomarkers for severity assessment.

The level of the biomarker CK correlated with the severity of the disease, although the HyperCKemia was found to be lower in COVID-19 than in influenza [76]. Elevated levels of 3-hydroxyisovaleric in serum have been reported in patients with severe COVID-19, likely due to enhanced SkM catabolism [77]. In animal models, lower levels of myo-inositol have also been demonstrated and suggested as therapeutic approach in COVID-19. In fact, myo-inositol metabolism impairment has been related to several chronic diseases, including metabolic syndrome, dyslipidemia, diabetes, and cancer [78]. The identification of indexes of disease severity has involved not only biological markers but also muscle measurements. Notably, some authors have proposed that measurements obtained by chest-computed tomography of the pectoralis muscle might predict disease severity and mortality rate of COVID-19 pneumonia in adult patients [79,80]. Contrarily, SkM index at the level of the 12th thoracic vertebra was not associated with negative outcomes in hospitalized patients [81].

### 2.2. Long Persistent Manifestations

Many survivors from severe COVID-19 have persistent manifestations long after resolution of the active infection (long COVID-19) [7,8,9,10,82]. The most commonly but highly variable reported physical health problems were fatigue [83], pain (myalgia, arthralgia), reduced physical capacity, and declines in physical role functioning, usual care, and daily activities (reduced in 15–54% of patients) [84]. Karaarslan et al., (2021) [84] reported that about 43% of patients had at least one musculoskeletal symptom, most frequently fatigue (32%), myalgia (15%), and joint pain (19%) at 6 months followed by back pain, low back pain, and neck pain. Similar variability was also found in common mental health problems such as anxiety, depression, and post-traumatic stress disorders [85,86]. Persistent dyspnea and fatigue, handgrip and quadriceps weakness [87,88], and reduction in diaphragm functionality in 76% of the patients have been described [89]. In some studies, gender variability was also observed. Indeed, greater fatigue, pain, anxiety, and depression were reported in female patients and individuals admitted to intensive care [90].

Several studies have reported that patients without previous locomotor disabilities show impairments in SkM strength and physical performance when recovering from COVID-19 pneumonia [91,92,93]. SARS-CoV-2 infection as well as a prolonged hospital stay might exacerbate sarcopenia since both inflammation and reduced exercise might increase SkM loss [18,94]. In addition to immobilization, dietary intake, aging, and low 25OH-vitamin D3 levels were inversely correlated with high IL-6 levels, and could lead to anabolic resistance and acute sarcopenia; thus favoring the disease severity and mortality [95]. Karaarslan et al., (2021, 2022) [84,96] also highlighted the possibility that COVID-19-related muscular injury might cause long-term disabilities. Of interest, patients who were hospitalized for two weeks manifested 32% and 13% reductions in grip strength and 3–6 months after discharge from the hospital, they still showed a poor health-related quality of life [97]. Notably, COVID-19 can aggravate preexisting neuromuscular diseases, such as myasthenia gravis [82] and strong infections can promote myopathy/polyneuropathy [19,97] (see below, Section 4).

## 3. Mechanisms of SARS-CoV-2 Infection of Skeletal Muscle Damage

The exact dynamics and molecular mechanism of SkM damage, as well as the long-term effects of this tissue injury in COVID-19 patients, are unclear. As described before, myalgia [20] and fatigue are frequent manifestations of COVID-19. Neuronal demyelination, which may contribute to muscle weakness and fatigue was also described for SARS [98]. A rapid decrease in body mass within 4 days of infection was also found in a mouse model of SARS [99]. SkM damage might be caused directly by viral infection as in alphavirus-induced myositis [100] or indirectly through inflammation (cytokine storm), autoimmune processes, or as consequence of myotoxic drugs.

### 3.1. Direct Effect: SARS-CoV-2 Infection of Skeletal Muscle Cells

Myalgia, muscular weakness, and fatigue could be the effect of direct invasion of myocytes by the virus, but also of inflammation-mediated injury. Other viruses such as influenza viruses, human immunodeficiency virus, enteroviruses, parainfluenza virus, and adenovirus are capable of promoting myopathies, including necrotizing myopathies [101]. Widespread muscle fiber atrophy was also described, with sporadic and focal muscle fiber necrosis in SARS-affected patients [16,99]. Up to now, SkM invasion by SARS-CoV-2 has not been consistently demonstrated although SkM cells express both TMPRSS2, the protease that facilitates the virus–cell fusion, and ACE2 even if at a low level compared to ACE2 in vasculature [102]. Dalakas et al., (2020) [103] suggested that SARS-CoV-2 might be the first virus capable of infecting muscle fibers directly and Ferrandi et al., (2020) [104] suggested that myopathy in COVID-19 could be the result of SARS-CoV-2 infection via ACE2. Several studies reported the presence of viral particles by electron microscopy, but these identifications were subject of criticism [105,106]. SARS-CoV-2 mRNA was detected by reverse transcription and real time polymerase chain reaction, but immunohistochemistry and electron microscopy analyses did not provide any evidence for a direct viral infection of myofibers [66,107,108]. Similarly, SkM atrophy and necrotizing myopathy have been reported in autoptic specimens from COVID-19 patients, without any detection of virus in the tissue [109]. In several reports, in which no evidence of SkM infection was found, a vascular localization of SARS-CoV-2 was proposed [69,71,110]. Therefore, the low viral load could be attributable to circulating viral RNA rather than direct infection of myocytes.

ACE2 belongs to the renin–angiotensin system (RAS) in humans. Classical and non-classical RAS pathways have been described [111]. The RAS pathway activation increases reactive oxygen species formation and protein degradation, and contributes to adverse consequences in SkM, via the transcription of the ubiquitin ligase TRIM63/MuRF-1 [112,113]. Therefore, SkM catabolism markers, such as TRIM63/MuRF-1, might predict disease severity. However, ACE2 is also involved in the known “non-classical RAS pathway,” which mainly involves the Angiotensin 1–7/Mas axis and contributes to anti-atrophic, anti-fibrotic, and anti-inflammatory activities in SkM. Up to now, it is not clear whether the increase in the classical RAS axis prevails over the decrease in the non-classical one in COVID-19.

Moreover, it is also reported that ACE2 functions through pathways outside of the RAS [114,115] involving either a peptidase-dependent or -independent pathway. The first is mainly due to the catalytic activity of ACE2, on specific targets, such as apelin-13, a crucial regulator in ACE2 gene expression [116], which may be worth investigating in SARS-CoV-2 infection.

### 3.2. Indirect Effect: Cytokine Storm and SkM Remodeling

Inflammation is an important contributor to the pathology of many tissues including SkM. In fact, a certain number of disorders including inflammatory myopathies are characterized by chronic inflammation or elevation of the pro-inflammatory mediators [117,118]. Several of the pro-inflammatory molecules increased in patients with COVID-19 negatively affect SkM. In fact, many of the components of the cytokine storm described in COVID-19 such as IFN-γ, IL-1β, IL-6, IL-17, and TNF-α can directly induce SkM fiber proteolysis and decrease protein synthesis. Moreover, IL-1β and TNF-α can block the proliferation and differentiation of satellite cells, which are the progenitor cells of muscle fibers crucial for muscle regeneration following muscle damage. Moreover, IL-1β and IL-6 can induce muscular fibrosis, which could impair muscle force generation [119]. SARS-CoV-2 hyper-inflammatory response contributes not only myofibrillar breakdown and degradation with progressive loss of SkM mass but also to immunosenescence, enhancement of the endothelial damage, and tissue weakness [120]. This hyper-catabolism, especially in aged people, is associated with oxidative stress, which further causes severe myocyte damage [121]. Inflammation was most pronounced in COVID-19 patients with chronic courses. Dysfunction in SkM caused by cytokine storm may lead to additional symptoms, such as shortness of breath, difficulty in swallowing and speaking, heart arrhythmias, and fatigue. Moreover, SkM dysfunctions in COVID-19 patients affected by inflammatory disorders such as diabetes, obesity, cardiovascular diseases, cancer, etc., are associated with more severe COVID-19 [7,8,9,10].

Interestingly, SkM complications in COVID-9 patients may also be due to adverse effects of several drugs used to counteract the disease. For example, it has been reported that SARS-CoV-2-associated rhabdomyolysis is rarely due to virus infection and most likely a consequence of anti-COVID-19 drug toxicity [122]. Additionally, corticosteroids, frequently used to control acute inflammation in patients with SARS, can directly promote muscle atrophy and weakness [123].

## 4. COVID-19 May Be a Risk for Patients Affected by Neuromuscular Diseases

It has been hypothesized that patients with chronic neuromuscular disorders may have an increased risk of developing severe symptoms of COVID-19. It is known that patients with dystrophinopathies or with motor neuron disease experience respiratory muscle weakness and cardiomyopathy that could be exacerbated following SARS-CoV-2 infection [124]. Although this is a plausible hypothesis, evidence that complications or mortality rates in neuromuscular patients are higher than in the general population has not been found in several studies [125,126,127,128,129,130].

### 4.1. Myasthenia Gravis

Myasthenia gravis (MG) is an autoimmune disease of the neuromuscular junction synapse characterized by weakness that worsens with continued muscle work and improves with resting of the involved muscle(s). Acetylcholine receptor antibodies are found in 90% of patients with generalized MG, whereas muscle-specific kinase (MuSK) antibodies are found in approximately 4% of cases [131]. MG disease may lead patients to be vulnerable to SARS-CoV-2 infection mainly due to respiratory muscle weakness, and long-term immunosuppressive treatment. In fact, studies have reported that although the rate of SARS-CoV-2 infection was comparable to that of the general population, the risk of hospitalization and death for MG patients was greater [132,133,134]. Contrarily, several reports indicate that the virus infection has variable effects (mild, exacerbation, or a fatal outcome) [109,135] that in patients treated with immunosuppressive drugs, such as MG patients, the outcome is favorable [136,137,138,139].

Very recently, Jakubíková et al., (2021) [140] have reported that long-term chronic corticosteroid treatment, especially at a high dose, worsened the course of COVID-19 in MG patients. On the other hand, MG may appear following COVID-19 infection, and, besides just coincidence, it could be hypothesized that SARS-CoV-2 could trigger MG and that molecular mimicry or latent MG activation could be involved in the onset of the disease [141,142,143]. Several cases of MuSK Antibody-Associated Myasthenia Gravis with SARS-CoV-2 infection have been reported [144,145]. In addition, a case of COVID-19 vaccine causing a MG crisis has been recently observed [146,147,148,149].

Notably, pretreatment of rats with Fingolimod improved experimental autoimmune MG symptoms in a dose-dependent manner, including decreased anti-acetylcholine receptor-2α autoantibody titer, reduced compound SkM action potential decrement, and increased acetylcholine receptor content. Fingolimod suppressed the secretion of pro-inflammatory or inflammatory cytokines IL-17A, IL-6, and INF-γ, without modifying the release of the immunosuppressive TGF-β1 and IL-4 [150]. Prophylactic administration of Fingolimod was also found to attenuate MG in rats by reducing the number of dendritic cells, follicular T helper cells, and antibody-secreting cells [151]. However, other authors did not find improvement in experimental autoimmune MG in mice when a lower dose of Fingolimod was administered after disease onset [152]. Since not only the animal model but also both the concentration and time of administration of the drug were different, it is unclear whether Fingolimod might have a preventive or a therapeutic effect in MG. Fingolimod is an analogue of sphingosine, which can be phosphorylated by sphingosine kinase (SphK) 2, generating a molecule similar to S1P and able to bind to S1PRs, except S1P2R. Fingolimod has been approved for the treatment of multiple sclerosis (MS), due to its action as an immunosuppressant. Indeed, it acts as a highly potent functional antagonist of the S1P1 receptor, promoting S1P1 receptor internalization in T cells that become unable to exit from the lymph nodes [153].

### 4.2. Multiple Sclerosis

MS is an immune-mediated disorder of the central nervous system, which is characterized by demyelination and axonal degeneration, which, consequently, contribute to SkM weakness.

MS disease onset and MS relapse after SARS-CoV2-infection have been reported [154,155,156] and it has been hypothesized that the inflammatory response to viral infection could contribute to both outcomes [156]. Some studies point to a higher risk of exacerbation after SARS-CoV-2 infection [157], but others have not found any difference [158] or proposed the necessity of more controlled studies [159].

Immunosuppressive MS therapies, such as Fingolimod, may modify the risk of developing a severe SARS-CoV-2 infection due to its action on the egress of lymphocytes from lymph nodes. However, several studies in different countries have demonstrated that most cases are found to have mild course and full recovery on disease modifying therapies [160,161,162,163,164]. Interestingly, it has been reported that a significant proportion of convalescent COVID-19 patients treated with natalizumab, fingolimod, alemtuzumab, ocrelizumab, cladribine, and ublituximab did not develop IgG SARS-CoV-2 antibodies [165,166,167,168]. It is possible that the therapies could decrease the SARS-CoV-2-induced cytokine storm.

Most COVID-19 patients develop an immunoglobulin G-SARS-CoV-2 antibody response, whose protective level is maintained up to nine months [169]. Moreover, 82.9% of patients show positive immunoglobulin G levels one year after infection [170]. The low levels of antibodies in MS patients could become a risk after reinfection and some authors recommend vaccination as soon as possible [171].

### 4.3. Amyotrophic Lateral Sclerosis (ALS)

ALS is a neurodegenerative disorder with progressive degeneration and death of upper and lower motor neurons, severe muscle atrophy, respiratory distress, and cellular protein aggregation [172]. Although muscle dysfunction has been considered a consequence of deprivation of innervation, evidence has accumulated indicating that alteration of SkM precedes motor neuron denervation and onset of ALS symptoms. Therefore, muscle could initiate and/or contribute to the cascade of pathological events [173].

At present, accurate data on SARS-CoV-2 infection in ALS patients is not available. One paper has studied the impact of COVID-19 among hospitalized ALS patients, but the number of patients was small (19) and only 6 patients tested positive [174], making it impossible to generalize the results. Bertran Recasens et al., (2020) [175] have studied the impact of COVID-19 on a cohort of ALS patients in Catalonia and they have not found an increase in mortality comparing the lockdown period with the same period of time before the pandemic. By contrast, Galea et al., (2021) [176] have reported that USA veterans with ALS were 3-fold more likely to die within 30 days of COVID-19 diagnosis compared to the overall veteran population.

A large non-coding hexanucleotide repeat expansion in the C9orf72 gene is the main genetic cause of frontotemporal dementia and ALS. Intermediate hexa-nucleotide repeats (>10 units) were found in only a small portion of 240 patients with severe COVID-19 pneumonia, but in these patients, the risk of requiring non-invasive or mechanical ventilation was more than twice with respect to the patients of a similar age having shorter expansions [177]. Interestingly, it has recently been reported that two patients with slowly progressive ALS showed a fast functional decline after contracting COVID-19 [178].

## 5. Sphingolipids as Biomarkers and Mediators of Virus Pathogenicity

### 5.1. Sphingolipid Metabolism and Sphingosine 1-Phosphate-Mediated Signaling

SLs are major constituents of membrane lipids and are enriched in microdomains. The modifications in their concentration influence membrane dynamics and trigger signaling events [27,179,180,181,182,183].

Sphingomyelin, the more concentrated SL in the plasma membrane of mammalian cells, can be hydrolyzed by sphingomyelinases generating phosphocholine and ceramide (Figure 1A,B). The de novo synthesis of SLs starts from serine and palmitate condensation promoted by serine palmitoyltransferase. Reduction and acylation of the product, 3-keto-dihydrosphingosine, produces dihydroceramide, and is then reduced to ceramide. Ceramide can also be phosphorylated to ceramide 1-phosphate and it is a substrate of ceramidases. The product of ceramidases is sphingosine, which can be phosphorylated by two SphK isoforms (SphK1 and SphK2) to generate the bioactive lipid S1P. Sphingosine can also be produced by S1P dephosphorylation by S1P phosphatase and lipid phosphate phosphatases. S1P is irreversibly degraded to hexadecenal and ethanolamine phosphate by S1P lyase. Notably, the interplay and the ratio between simple SLs, in particular S1P, ceramide, and sphingosine, is a crucial determinant of cell fate. In fact, ceramide and sphingosine can control cell growth arrest, cell stress, senescence, and apoptotic death, whereas S1P and ceramide 1-phosphate regulate cell proliferation, migration, and survival [27,37]. S1P has been shown to prevent programmed cell death also induced by ceramide. S1P can act either as an intracellular mediator and a ligand of specific heterotrimeric GTP binding protein-coupled receptors, S1PRs, leading to specific multiple responses [35,37,184]. S1P can be also released outside the cells by specific transporters belonging to the Major Facilitator Superfamily, such as Spinster 2 (Spns 2) and Mfsd2b [32,185] (Figure 1C,D). This homeostatic system, known as the ceramide/S1P rheostat, functions also in SkM cells: in many cases, the increase in S1P level displays largely positive actions, whereas intracellular accumulation of ceramides exerts opposite roles.

### 5.2. Sphingolipids and Skeletal Muscle Remodeling

SLs are able to modulate SkM cell proliferation, differentiation, SkM mass and tissue regeneration [186]. An increase in acidic sphingomyelinases activity is associated to degradation of SkM and the decrease in SkM force in mice [187], likely associated also with aging [188]. In addition, it has been reported that the inhibition of acidic sphingomyelinases activity improved SkM insulin response of old rats reaching the level of younger rats [189], supporting a role of acidic sphingomyelinases in age-dependent SkM pathologies. Regarding the S1P/S1PR axis, the trophic action of S1P has been reported in in vivo and in vitro studies. S1P positively affects skeletal muscle growth and differentiation in the C2C12 muscle cell line, derived from mouse muscle stem cells, as well as in satellite cells [43,47,190]. S1P formation by SphK1 contributes to the cell cycle arrest and promotes the myogenesis, while the inhibition of SphK1 expression increases myoblast proliferation and delays cell differentiation [43,191]. 

Interestingly, S1PR pattern expression changes during myoblast differentiation into myotubes. Indeed, a downregulation of the S1PR2, and an upregulation of S1PR3 in differentiated myotubes have been observed [41]. In addition to muscle differentiation, S1P plays an important role in skeletal muscle regeneration. S1P stimulates the growth of regenerating myofibers after a myotoxic injury induced by intramuscular injection of bupivacaine [51], suppresses muscle degeneration in Duchenne muscular dystrophy [192], and exerts a positive action for muscle regeneration in dystrophic muscles [193,194]. S1P could protect SkM tissue against eccentric contraction-induced damage, further underscoring the relevance of S1P signaling in SkM protection [43]. S1P through S1PR-mediated signaling modulates cell regeneration [40,41,43,46,51]. Indeed, Germinaro et al., (2012) [195] reported that the S1P/S1PR2 axis favors SkM regeneration and its absence completely inhibited the pro-myogenic functions of satellite cells, whereas activation of the S1P/S1PR3 signaling suppresses cell cycle progression in muscle satellite cells [45].

The importance of S1P/S1PR in controlling the SkM phenotype has also been highlighted in [46]. In fact, the impairment in S1PR2 and S1PR3 expression has been observed in SkM tissue from cachectic mice as well as dexamethasone-induced muscular atrophy in C2C12 cells. In the latter system, SphK and S1P transporter dysfunctions have been also demonstrated. Contrarily, ceramide appears to negatively control myogenesis as well as SkM mass. Indeed, TNFα-induced increase in ceramide level inhibited the myogenesis [52], while the inhibition of the de novo ceramide synthesis by myriocin increased the appearance of differentiated phenotype [53] and improved myocyte size in a mouse model of cancer-induced cachexia [54]. Recently, our group reported that a downregulation of ceramide kinase is associated with tumor-induced SkM mass wasting in cachectic mice and cell atrophy induced by dexamethasone [55]. All these considerations reinforce the action of the ceramide/S1P rheostat on SkM differentiation and muscle mass and fatigue regulation. Unfortunately, no human studies corroborating these results have been published so far. Furthermore, modifications of SL metabolism regulate both fatigue and strength of SkM [44,50,196]. Some data also suggested that SL metabolism could be involved in the development of sarcopenia with aging. Aging is characterized by an increase in intramyocellular lipid content. These lipid droplets, found in most eukaryotic cells, accumulate also in SkM cells likely contributing to cytoskeletal remodeling and SkM cell differentiation [197]. Rivas et al., (2016) [198] provided evidence of a significant increase in different classes of ceramide, sphingosine, and S1P in SkM cells from older mice compared to young animals. This SL accumulation associated with inflammatory processes, may contribute to mass decrease observed in SkM of aged mice [199].

Finally, in recent years, it has been demonstrated in several studies that exercise induces an important effect on SL metabolism [200,201,202]. In addition, SkM has emerged as a secretory tissue for cytokines, named myokines such as interleukin-6 (IL-6) released from the tissue in response to exercise [203,204,205,206].

### 5.3. Sphingolipids and Viral Replication

SARS-CoV-2 virus as well many other viruses require plasma membrane remodeling for cell infection. Therefore, SLs enriched in microdomains may serve as an “enhancer” of virus infection by controlling virus receptor availability/segregation, allowing fusion at the plasma membrane and triggering endocytosis-mediated uptake and virus release [29,207,208,209]. SARS-CoV-2 binds to ACE2, but also to sialic acids of gangliosides [210]. In fact, the N-terminal domain of the Spike protein has a ganglioside-binding domain that enables the virus to bind to the lipid rafts of the plasma membrane, where the ACE2 receptor is located [211]. This is also of interest since Coronaviruses, like other positive-sense RNA viruses, remodel the intracellular membranes to form specialized viral replication compartments, such as double-membrane vesicles, where viral RNA genome replication takes place [212,213]. In particular, the sphingomyelinase, by producing ceramide, converts membrane rafts into ceramide-enriched platforms, which are sites of endocytic uptake of pathogens, due to concentration of their receptors, or formation of adaptors and signaling complexes [214,215,216]. Consequently, well-known inhibitors of the sphingomyelinase activity represent promising COVID-19 therapeutic approaches [29,217,218,219,220].

Furthermore, viruses can also use the host lipid machinery to support their life cycle and to impair the host immune response [221]. In particular, SARS-CoV-2 infection promotes remodeling of the host cell metabolism, including lipid metabolism [222,223].

**Figure 2:**
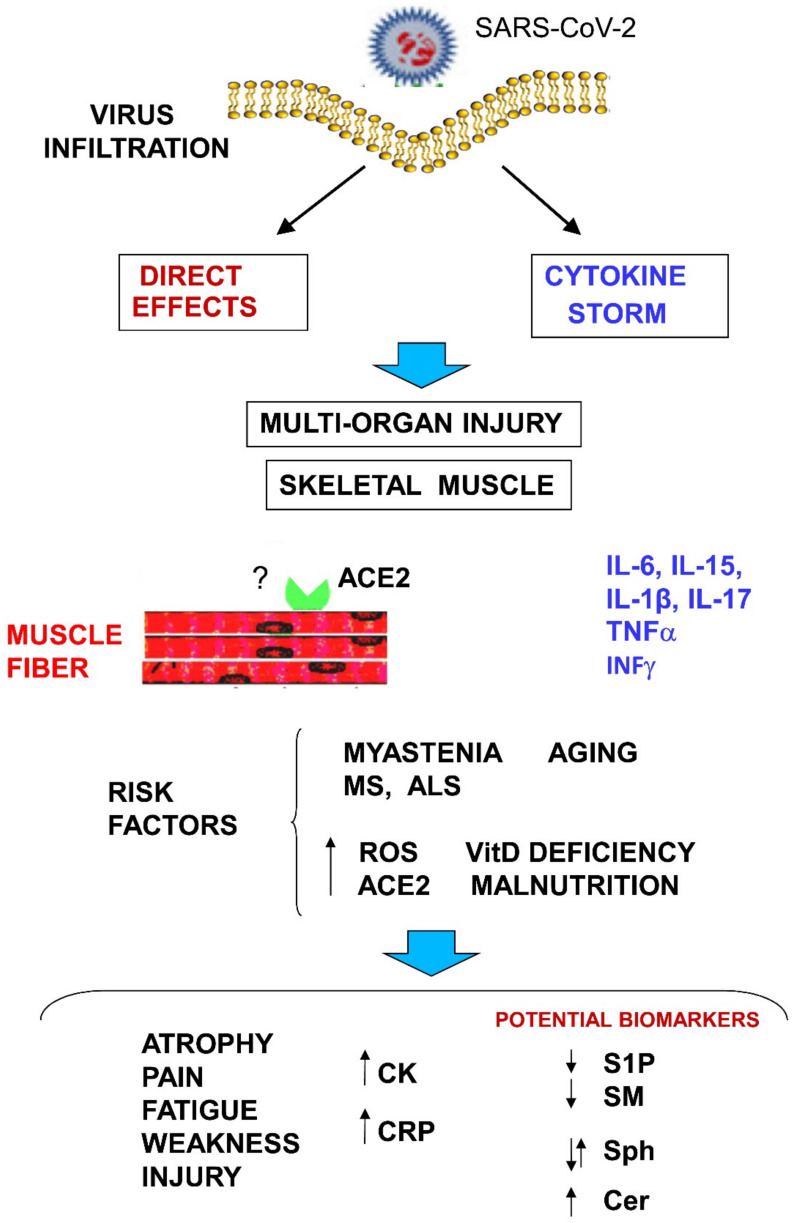
Potential effects, risk factors, and potential biomarkers of SARS-CoV-2 infection into skeletal muscle. Viral infection or cytokine storm can lead to multiorgan injury, including skeletal muscle damage through multiple pathways (see text). Risk factors for developing COVID-19 are reported. They include aging, malnutrition, vitamin D3 deficiency, neuromuscular diseases such as myasthenia gravis, multiple sclerosis (MS) and amyotrophic lateral sclerosis (ALS), high expression of ACE-2, or high levels of reactive oxygen species (ROS). Skeletal muscle symptoms of damage could lead to increase in typical biochemical markers of tissue damage and inflammation such as creatine kinase (CK) and protein C-reactive (PCR). Several sphingolipid metabolites and enzymes have been proposed as biochemical markers. Ceramide (Cer), sphingosine (Sph), sphingosine 1-phosphate (S1P), ceramide synthase (CerS), and sphingomyelin (SM). ? indicates that direct infection of SARS-CoV-2 into muscle skeletal cells is still to be demonstrated. VitD, Vitamin D.

In order to better predict the progression of the disease, many studies have investigated the correlation between clinical features and lipidomics, metabolomics, and proteomic profile in COVID-19-patients [224]. Thomas et al., (2021) [223] reported significant decreases in sphingosines and increases in ceramide–phosphorylethanolamine as the most affected classes in red blood cells from COVID-19 patients, while in a lung cell line infected with SARS-CoV-2. Moolamalla et al., (2021) [222] have found increased expression of the genes encoding for sphingomyelin synthases 1 and 2, serine palmitoyltransferase long chain base subunits 1 and 2, and ceramide synthase isoforms 2, 5, and 6, as well as several genes involved in glucosphingolid synthesis. This is interesting since it has been recently reported that glucosylceramide synthase inhibitors prevent replication of SARS-CoV-2 and influenza virus [225]. Moreover, Janneh et al., (2021) [226] have provided evidence that levels of acid ceramidase are increased in serum of 73% of asymptomatic patients and that reduced sphingosine levels could constitute a sensitive biomarker for the development of symptomatic COVID-19 likely due to its inhibitory action in the binding of Spike protein to ACE2 [227]. A correlation between serum S1P and SARS-CoV-2 infection has also been observed. S1P and S1P-metabolizing enzymes such as S1P lyase and SphK have been shown to regulate viral functions during infection [228] and in sepsis [229], by interfering with innate immune responses. Torretta et al., (2021) [230] reported also the decrease in sphingomyelin and S1P levels in severe COVID-19 patients. Very recently, the level of circulating S1P has been suggested to be of clinical importance as a prognostic and predictive biomarker in COVID-19 disease [231]. In fact, serum S1P and apoM appear associated with COVID-19 severity and morbidity, due to their role in endothelial barrier dysfunctions, altered immune response, and persistent excessive inflammation in COVID-19 patients. Moreover, since sphingomyelin concentration is correlated to the inflammatory processes and viral infection of the respiratory tract, two classes of sphingomyelins (d18:2/20:0 and d18:1/22:2) have been suggested as candidate biomarkers to monitor disease progression and severity [232]. In another study of lipidomic analysis of the plasma samples obtained from 52 COVID-19 infected individuals it has been reported that ceramide exhibited a 400-fold increase in the infected patients. Notably, the ceramide species Cer(d18:0/24:1), Cer(d18:1/24:1), and Cer(d18:1/22:0) were increased by 48-, 40-, and 33-fold, respectively, and to 116-, 91-, and 50-fold in plasma samples of patients with respiratory distress [36]. Other authors have described that patients with severe disease showed progressive increase in sphingosine, dihydrosphingosine, dihydroceramides, ceramides, but not of glycosphingolipids, suggesting that also ceramides C16:0, C18:0, and C24:1, may be putative biomarkers of disease evolution and respiratory symptoms severity [230] (Figure 2).

## 6. Future Directions

Recent studies have uncovered the dysregulation of metabolomic and lipidomic profiles of COVID-19 patients [233,234,235] and were oriented to find a correlation with disease progression and severity. SL metabolism was found to be one of the more involved pathways in COVID-19 patients [36,230,231,232,236,237,238,239,240,241,242].

These lipidomic/metabolomic studies were performed in serum of COVID 19 patients, but no data are available from different tissues involved in this multi-organ disease. Thus, a comprehensive lipidomic analysis of SkM tissue and a deep investigation of specific subclasses of sphingoid molecules, SL metabolism, and S1P signaling in SkM tissue may be of crucial importance for the identification of pharmacological strategies directed to these potential and exciting targets. Moreover, both SkM disuse and sarcopenia are factors that can increase the risk of developing acute and long-term dysfunctions observed in COVID-19 patients. SkM inactivity and aging are correlated with SL- and cholesterol-enriched membrane microdomain remodeling, changes in ceramide pools [243] and S1P/S1PR signaling [186,244]. Notably, it could be interesting to examine the effects of the inhibitors of acid sphingomyelinase on SkM, since these inhibitors are promising therapeutic drugs against COVID-19 [245,246] and, likely, long COVID-19. Further investigations will certainly be necessary to clarify the complex functional relationship between the action of bioactive sphingoid molecules and SkM dysfunctions in COVID-19 patients.

## Data Availability

Data sharing not applicable.
